# Algorithmic Annotation of Functional Roles for Components of 3,044 Human Molecular Pathways

**DOI:** 10.3389/fgene.2021.617059

**Published:** 2021-02-09

**Authors:** Maxim Sorokin, Nicolas Borisov, Denis Kuzmin, Alexander Gudkov, Marianna Zolotovskaia, Andrew Garazha, Anton Buzdin

**Affiliations:** ^1^Omicsway Corp., Walnut, CA, United States; ^2^Laboratory of Clinical Genomic Bioinformatics, I.M. Sechenov First Moscow State Medical University, Moscow, Russia; ^3^Laboratory for Translational Bioinformatics, Moscow Institute of Physics and Technology, Moscow, Russia; ^4^Laboratory of Systems Biology, Shemyakin-Ovchinnikov Institute of Bioorganic Chemistry, Moscow, Russia; ^5^World-Class Research Center “Digital Biodesign and Personalized Healthcare”, Sechenov First Moscow State Medical University, Moscow, Russia

**Keywords:** functional algorithmic annotation, signaling pathways, DNA repair pathways, metabolic pathways, transcriptomics, proteomics, human molecular pathway regulation

## Abstract

Current methods of high-throughput molecular and genomic analyses enabled to reconstruct thousands of human molecular pathways. Knowledge of molecular pathways structure and architecture taken along with the gene expression data can help interrogating the pathway activation levels (PALs) using different bioinformatic algorithms. In turn, the pathway activation profiles can characterize molecular processes, which are differentially regulated and give numeric characteristics of the extent of their activation or inhibition. However, different pathway nodes may have different functions toward overall pathway regulation, and calculation of PAL requires knowledge of molecular function of every node in the pathway in terms of its activator or inhibitory role. Thus, high-throughput annotation of functional roles of pathway nodes is required for the comprehensive analysis of the pathway activation profiles. We proposed an algorithm that identifies functional roles of the pathway components and applied it to annotate 3,044 human molecular pathways extracted from the Biocarta, Reactome, KEGG, Qiagen Pathway Central, NCI, and HumanCYC databases and including 9,022 gene products. The resulting knowledgebase can be applied for the direct calculation of the PALs and establishing large scale profiles of the signaling, metabolic, and DNA repair pathway regulation using high throughput gene expression data. We also provide a bioinformatic tool for PAL data calculations using the current pathway knowledgebase.

## Introduction

Intracellular molecular pathways are specific networks of interacting molecules that are involved in certain molecular functions (Junaid et al., 2020; Ma and Liao, 2020; Zheng et al., 2020). Knowledge of molecular pathways regulation is important for understanding intracellular processes related to all major events, including cell survival, growth, differentiation, motility, proliferation, senescence, malignization, and death (Buzdin et al., 2018). Molecular pathways are affected during organism growth and development, aging and disease progression (Parkhitko et al., 2020). Current methods of large-scale molecular and genomic analyses enabled to catalogue thousands of human molecular pathways (Wishart et al., 2020). In turn, high-throughput gene expression analyses like RNA sequencing (Sorokin et al., 2020a), expression microarrays (Schulze and Downward, 2001; Shih et al., 2005; Willier et al., 2013), or modern proteomic techniques (Buzdin et al., 2019) can provide adequate amounts of data to enable interactome-wide assessment of pathway activation.

Several popular algorithms and software like gene ontology (GO) analysis tools (Huang et al., 2009a,b), Metacore (Ekins et al., 2007) and Pathway Studio (Thomas and Bonchev, 2010) can analyze gene expression data to identify pathways significantly enriched by differentially regulated genes (Dubovenko et al., 2017). However, those techniques cannot identify the enhanced or inhibited status of a pathway regulation, because pathways may have numerous negative feedback loops or negative regulatory nodes (Khatri et al., 2012) and, therefore, the pathway nodes may involve both genes with its activating and genes with inhibitory functions (Borisov et al., 2020). Thus, upregulation of an inhibitory gene means pathway downregulation, and vice versa (Buzdin et al., 2018).

On the other hand, knowledge of the individual gene product roles within a pathway can make it readable in terms of finding its activation profiles. Indeed, several techniques had been proposed, e.g., Oncofinder (Buzdin et al., 2014b), iPANDA (Ozerov et al., 2016), and Oncobox (Borisov et al., 2020) that utilize transcriptome-wide or even proteome-wide (Borisov et al., 2017) data to calculate pathway activation levels (PALs). Those are the numeric characteristics that can be used in all types of comparisons including biomarker investigations. Overall, PALs were found to be superior cancer biomarkers compared to individual gene expression levels (Borisov et al., 2014; Lezhnina et al., 2014). A number of PALs were found to be characteristic for cancer drug response (Zhu et al., 2015) and sensitivity to X-ray irradiation (Sorokin et al., 2018), asthma (Alexandrova et al., 2016), Hutchinson-Gilford progeria (Aliper et al., 2015), macular degeneration (Makarev et al., 2014), fibrosis (Makarev et al., 2016), viral infection (Buzdin et al., 2016), and aging (Aliper et al., 2016). Algorithms were developed to convert pathway activation data into the optimized selection of cancer drugs (Artemov et al., 2015; Tkachev et al., 2020) that had several recent clinical applications (Poddubskaya et al., 2018, 2019a,b; Sorokin et al., 2020b). However, those studies used manually curated/annotated pathways and were, therefore, limited by the overall number (~10 or ~100) of pathways under analysis. Thus, it is important to annotate more pathways in a universal way to obtain a large-scale overview of the human interactome.

We proposed an algorithm that identifies functional roles of the pathway components based on the pathway topology and applied it here to annotate 3,044 human molecular pathways extracted from the Biocarta, Reactome, KEGG, NCI, and HumanCYC databases, collectively covering 9,022 gene products. The resulting knowledgebase can be applied for the direct calculation of the PALs and establishing large scale profiles of the signaling, metabolic, and DNA repair pathway regulation using high throughput gene expression data.

## Results and Methods

### Extraction of Molecular Pathway Data

We extracted structures of molecular pathways from the National Cancer Institute (NCI; Schaefer et al., 2009), Biocarta (Nishimura, 2001), Qiagen Pathway Central,^1^ HumanCyC (Romero et al., 2004), Reactome (Croft et al., 2014), and Kyoto Encyclopedia of Genes and Genomes (KEGG; Kanehisa et al., 2010) databases (Table 1). For all the databases but Qiagen Pathway Central, the data on the pathway architecture, nodes and pairwise activation/inhibition interactions were extracted in *biopax* format. In the case of Qiagen Pathway Central database, no machine-readable format of data was available, and we manually curated data from the available graphical pathway representations (Table 1).

**Table 1:** Statistics of the curated pathway databases.

Database	References	Number of			Data curation format
		core pathways	all pathways	unique genes	
Biocarta	Nishimura, 2001	198	337	1,082	Automated
Reactome	Croft et al., 2014	945	945	6,105	Automated
KEGG	Kanehisa et al., 2010	288	288	5,593	Automated
Qiagen	Pathway Map Reference Guide–QIAGEN, 2014	57	380	2,493	Manual
NCI	Schaefer et al., 2009	211	775	2,214	Automated
HumanCYC	Romero et al., 2004	319	319	1,038	Automated
Total number		2,018	3,044	9,022	

Number of all pathways includes core pathways and micropathways. Number of unique gene products covered by pathways from the respective database. For total number, the amount of unique gene products for all pathways is shown.

**Figure 1:**
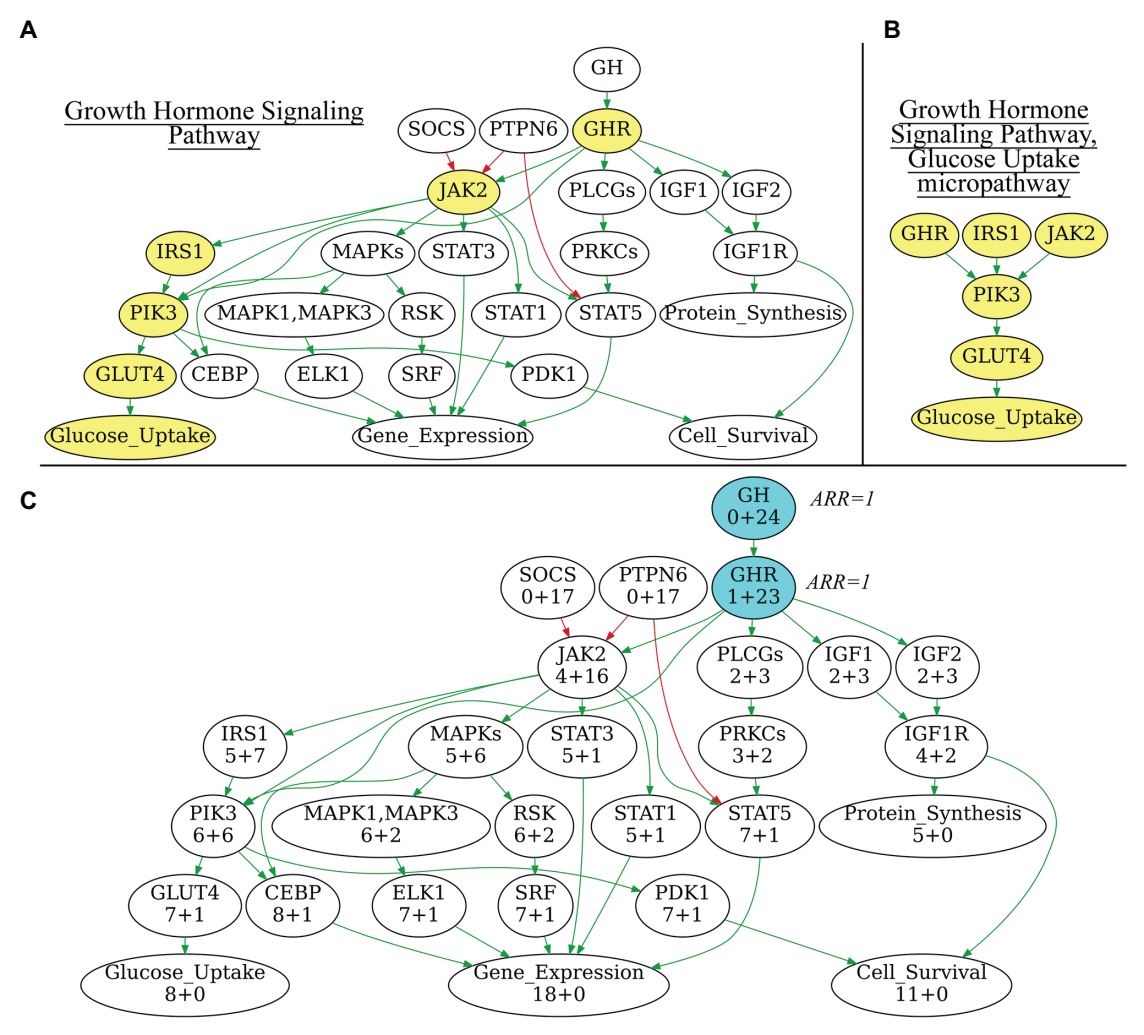
**(A)** Growth Hormone Signaling Pathway with highlighted Glucose Uptake micropathway. **(B)** Glucose Uptake micropathway obtained from Growth Hormone Signaling Pathway. **(C)** N+M values for all vertices of Growth Hormone Signaling Pathway graph. The vertices with maximal N+M values are highlighted in blue, these vertices are equal *major node* candidates and get Activator/Repressor Role (ARR) = 1. Different edge colors indicate edge attribute: green is for “activation,” red is for “inhibition.” Structure of the Growth Hormone Signaling Pathway is derived from Qiagen Pathway Central. Yellow vertices on panel 1A indicate micropathway Glucose Uptake within Growth Hormone Signaling Pathway.

In addition to the extracted full-size pathways, we also generated a number of subsequent “micropathways” that were derivatives of the complete pathways (Figures 1A,B) Micropathway is a sub-graph, which contains “molecular function” node and nodes from all possible paths of length 3 including terminal “molecular function” node. Many full-size pathways have two or more terminal branches that may have different functional impact(s). We, therefore, introduced micropathways to characterize molecular processes in more detail by separately analyzing different terminal branches of the pathways. Totally, we processed 3,044 pathways including 2018 full-size, or “core” pathways, and 1,026 micropathways that covered collectively products of 9,022 human genes (Table 1). Note that number of pathway nodes was smaller than the number of genes involved in a pathway because one node could correspond to several gene products.

For several pathway components alternative gene names were used and we then converted all gene names according to the Human Genome Organization HGNC nomenclature (Povey et al., 2001).

### Algorithmic Annotation of Molecular Pathways

For most of the published PAL applications, maximum five types of functional roles for gene products were comprised. These roles, described by an Activator/Repressor Role (ARR) parameter can be formulated as follows: pathway activator (ARR = 1), rather activator (ARR = 0.5), repressor (ARR = −1), rather repressor (ARR = −0.5), and gene product with uncertain or inconsistent role (ARR = 0). In the previous studies, ARR values were obtained by manually curating pathway graphs. This is however not feasible for annotating thousands of molecular pathways. We developed an original algorithm that automatically assigns ARR score values to gene products that participate in a molecular pathway.

The ARR annotation algorithm is based on the machine reading of gene product interaction graph within each pathway. Nodes correspond to gene products, and the ribs between every pair of nodes represent molecular interaction between the corresponding gene products. Each rib on the graph has a direction and is characterized by an activator or inhibitory nature of the molecular interaction it represents. For the correct calculation of ARR values, the pathway graph must be connected, wherein a weak connectivity is acceptable.

If the pathway molecular interaction graph meets these criteria, then ARR coefficients can be algorithmically assigned to the participating gene products. For the biochemical pathways, we put enzyme gene names on the pathway nodes, and the interaction ribs represented directions of the catalyzed reactions.

The algorithm used consisted of the following major steps.

- Initialization. At this stage, a major node is algorithmically identified to be the “central” node of the pathway graph (Figure 1C). The major node will be used as the standard of pathway function with ARR = 1. To identify the central node, for every pathway node (V) two parameters N and M are calculated where N is the number of other nodes, which can be reached when moving from the node V, and M is the number of other nodes from which the node V can be reached. N+M, therefore, is the number of other nodes that are directly connected with the node V. The central node will be the node V_max_ for which N+M reaches the maximum value. The central node identified is then assigned with ARR = 1 value. It serves as the starting point for further recursive assignment of ARR values to the other nodes. If multiple nodes have the same maximal N+M, then V-node for a pathway is selected randomly among those “maximal” nodes. Therefore, the algorithm is suitable also for circular-organized pathways, where all nodes will have equal N+M.
- Recursion. For every node V, all connected nodes P_i_ under ARR annotation may have ribs either directed toward V (Pi → V) or outward V (Pi ← V) on the graph. During recursion, each rib can be considered only once in order to prevent endless recursion in case of cyclic interactions on the graph. If the rib has an “activator” characteristic, temporary ARRtemp = 1 is assigned to the node P_i_. In contrast, if the rib has an “inhibitor” characteristic, P_i_ is assigned with ARR_temp_ = −1. Conversely, all the gene products included in the node P_i_ receive the same ARR_temp_ characteristics.

Let gene product GP_i_ belongs to node P_i_. If GP_i_ was never previously considered in the graph traversal, ARR = ARR_temp(Pi)_ for the node P_i_ would be assigned for GP_i_. In the case when GP_i_ was previously considered in the graph traversal and the previously assigned ARR of it node is equal to the current ARR_temp(Pi)_ then ARR = ARR_temp_ would be assigned to the node P_i_. If GP_i_ was previously considered in the graph traversal but its previously assigned ARR is not equal to ARR_temp(Pi)_, then ARR is assigned to the gene product GP_i_ according to the following conflict resolution rule.

If a gene product GP_i_ with previously specified ARR or ARRs is currently considered in the graph traversal but its previously assigned ARR(s) contradict(s) with the ARR_temp(Pi)_, then the conflict(s) should be resolved as follows:

1. If the signs of the previous ARR coefficient(s) and ARR_temp(Pi)_ are different, then the resulting ARR_final(Pi)_ = 0;
2. If the difference between ARR_temp(Pi)_ and any of the previous ARRs_(GPi)_ does not exceed 0.5 and at least one of the ARRs is positive, the resulting ARR_final(Pi)_ = 0.5;
3. If the difference between ARR_temp(Pi)_ and any of the previous ARRs_(GPi)_ does not exceed 0.5 and at least one of the ARRs is negative, the resulting ARR_final(Pi)_ = −0.5.

Then the recursion R is initiated for every node P_i_ all of its gene products starting from the nodes proximate to the central node V. As a result, the algorithm will assign ARR values to all the connected the graph nodes and the enclosed gene products.

After the recursion finalization pathway activators will have ARR = 1, rather activators – ARR = 0.5, inhibitors – ARR = −1, rather inhibitors – ARR = −0.5, and genes with inconsistent role – ARR = 0. The recursion is stopped when a vertex with 0, 0.5, or −0.5 ARR is encountered during the traversal of the graph. This rule is needed because otherwise all vertices will have ARR 0, 0.5, or −0.5 in case of the only one ARR inconsistency found. However, this rule also may lead to exclusion of some genes described in the original source.

Therefore, the gene products included in the molecular pathway database will have the assigned ARR values representing their functional significances in the given molecular pathway. These values can be used for further calculations of the PALs according to any algorithm of PAL calculation.

### Annotated Pathways Knowledgebase

We report here an ARR-curated database of 3,044 molecular pathways including 2,018 core pathways and 1,026 micropathways (Supplementary Dataset 1). The current pathway name reflects its source database and its name in the source database. For every pathway, there is a separate .csv file including the following three worksheets: (i) genes, (ii) edges, and (iii) nodes. The worksheet (i) *genes* include gene names according to HUGO Gene Nomenclature Committee (HGNC) nomenclature and the corresponding ARRs for the gene products participating in the pathway under consideration. The worksheet (ii) *edges* include information about molecular interactions between every pair of the interacting pathway nodes. Every node is defined by the names of gene products or physiological outcome(s) that form this node. The interaction type is specified as “activation,” “inhibition,” or “undefined,” where appropriate. The worksheet (iii) *nodes* include node names and gene names corresponding to every node on the pathway graph.

It should be noted that annotation of similar pathways may be different between the source databases. For example, EGFR signaling pathway is presented in Qiagen database as “EGF_Pathway,” in Reactome as “reactome_Signaling_by_EGFR_Main_Pathway” and in Biocarta as “biocarta_egf_signaling_Main_Pathway.” Yet conceptually similar, all three pathways have different gene and edge compositions. In this study, we did not aim to identify inconsistences between different source databases and annotated all the pathways under their original names.

We made freely accessible software for PAL calculation using the annotated pathway database accessible following the link: https://pypi.org/project/oncoboxlib/. Algorithm is implemented as a Python library. It takes normalized (by DESeq2, quantile normalization or other) gene expression data as an input. Gene symbols should be provided in HGNC format accessible through the web-site genenames.org. At least two groups of samples are required: cases and controls, each group represented by at least one sample. Sample names should contain “Norm_” (for controls) or “Tumour_” (for cases). Output will contain PAL values for each pathway in each sample. All annotated pathway datasets mentioned in this paper alternatively can be downloaded and used for PAL calculation using the same link.^2^

We also provide here an example of PAL calculation for real-world data. We extracted gene expression data for gastric cancer samples (*n* = 16; Sorokin et al., 2020b) together with gene expression profiles of healthy stomach (*n* = 7) samples of patients who died in road accidents (Suntsova et al., 2019), that were sequenced using the same equipment and protocols. Cancerous samples were marked as “Tumour_” and normal samples – as “Norm_.” Then we calculated PAL values (3,044 for each sample) for all molecular profiles using the above software, which produced an output file “pal.csv” (Supplementary Dataset 1).

## Discussion

**Figure 2:**
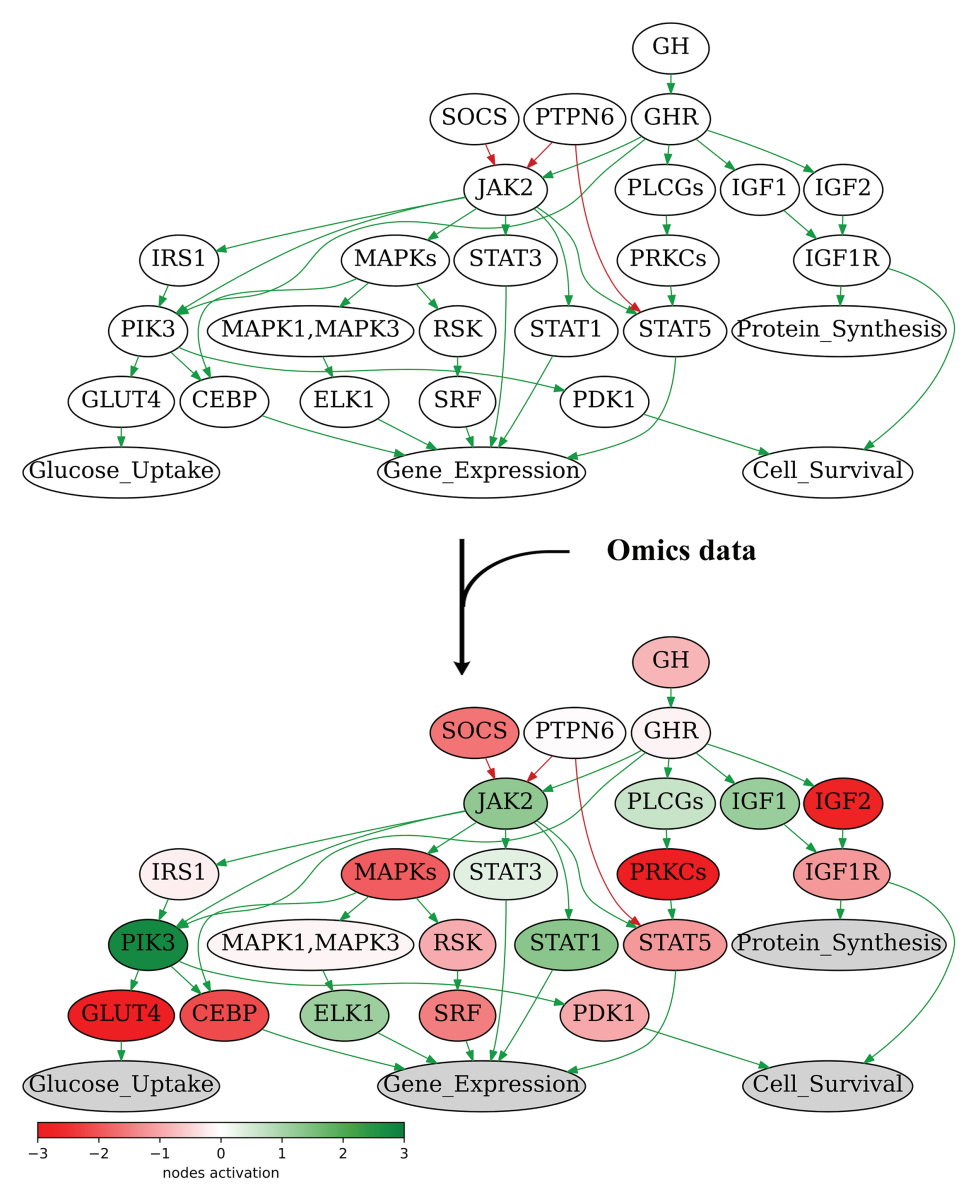
Node activation of Growth Hormone Signaling Pathway for gastric cancer sample GC.11_S19_R1_001 from Sorokin et al. (2020b). Node activation is a sum of logarithmic case-to-norm ratio (CNR) for all genes in the node. CNR is ratio of expression levels in tumor sample and averaged normal sample. The RNA sequencing tumor profile (gastric cancer) was obtained from Sorokin et al. (2020b). The RNA sequencing profiles of normal gastric tissue were obtained from Oncobox Atlas of Normal Tissue Expression (ANTE) data (Suntsova et al., 2019). Different edge colors indicate edge attribute: green is for “activation,” red is for “inhibition.” Structure of the Growth Hormone Signaling Pathway is derived from Qiagen Pathway Central.

We propose here the recursive algorithm for functional annotation of the molecular pathway nodes, and its application to annotation of 3,044 human molecular pathways, including signaling, metabolic, and DNA repair pathways extracted from six major pathway hubs (Table 1). The ARR-annotated pathways can be used for further calculations of PALs using high-throughput gene expression data, e.g., RNA sequencing or proteomic profiles (Buzdin et al., 2018; Figure 2). To this end, several previously published bioinformatic methods can be employed (Buzdin et al., 2014a; Ozerov et al., 2016; Borisov et al., 2020), and the PAL values returned can be applied for a variety of applications including fundamental research (Pasteuning-Vuhman et al., 2017), drug development (Aliper et al., 2017a; Ravi et al., 2018; Bakula et al., 2019), and personalized medicine (Poddubskaya et al., 2019a; Moisseev et al., 2020). Technically, PAL values can be used as the next-generation molecular biomarkers (Aliper et al., 2017b; Borisov et al., 2017; Sorokin et al., 2020c) or as the substrates for various machine learning applications (Borisov et al., 2018; Tkachev et al., 2018).

The proposed algorithm is suitable for the analysis of pathways with already established gene content and known topology of its molecular components. The algorithm can be used for agnostic objective characterization of interacting gene networks. The underlying rationale allows reducing operator’s errors and subjectivity in annotating the molecular roles of pathway components, which are inevitable in case of manual curation of the pathway graphs including hundreds of nodes. Another advantage is the pathway-centric approach during annotation, when gene product role in one pathway can be different from its role in another pathway.

The major limitations deal with the algorithm applicability only for the tasks of further calculations of pathway activation scores/ranks. Such an approach also does not address the issue of crosstalk between different molecular pathways, because all pathways are analyzed separately.

In this study, we annotated a collection of previously published human molecular pathways (Supplementary Dataset 1). We plan to update the current human knowledgebase annually with new releases of already included datasets and addition of new pathway collections, e.g., recently published by Wishart et al. (2020). However, the method proposed here can be used to characterize any new set of molecular pathways with the connectivity and pairwise nodes activation/inhibition information not only for the human interactome, but also for the other biological objects under investigation.

## Data Availability Statement

The original contributions presented in the study are included in the article/Supplementary Material, further inquiries can be directed to the corresponding author.

## Author Contributions

MS, NB, DK, and AB contributed to conception and design of the study. MS developed recursive pathway annotation algorithm. DK, AGu, MZ, and AGa manually curated the pathways and performed recursive algorithm implementations. AB, MZ, and MS wrote the manuscript. All authors contributed to the article and approved the submitted version.

### Conflict of Interest

MS, AGa, and AB have a financial relationship with OmicsWay Corp.The remaining authors declare that the research was conducted in the absence of any commercial or financial relationships that could be construed as a potential conflict of interest.The handling editor declared a shared affiliation with the authors AB, MS, and AGu at the time of review.

## Footnotes

^1^https://www.qiagen.com/gb/resources/resourcedetail?id=5869e38a-5033-4ccb-a281-d869893acf4e&lang=en

^2^https://pypi.org/project/oncoboxlib/
