# Supplementary material for: Algorithmic Annotation of Functional Roles for Components of 3,044 Human Molecular Pathways
Source: Front Genet. 2021 Feb 9;12:617059. doi: 10.3389/fgene.2021.617059 (PMC7900570; doi:10.3389/fgene.2021.617059)
Supplement: Supplementary file 2 [file Data_Sheet_2.pdf]

Supplementary Material

1 Supplementary Data

Supplementary dataset 1. ARR-curated database of 3044 molecular pathways including 2018 core pathways and 1026 micropathways.
